# Integrating CT radiomics and morphologic features for preoperative risk stratification of gastrointestinal stromal tumours

**DOI:** 10.1038/s41598-026-66765-x

**Published:** 2026-08-25

**Authors:** Fatmaelzahraa A. Denewar, Manar Mansour, Ahmed E. Eladl, Khadija Denewar, Adel Denewer, Ahmed Abdallah, Gehad A. Saleh

**Affiliations:** 1 Department of Radiology, Faculty of Medicine, Mansoura University, Mansoura, 35516 Egypt; 2 Department of Pathology, Faculty of Medicine, Mansoura University, Mansoura, Egypt; 3 Public Health and Community Medicine Department, Faculty of medicine, Mansoura University, Mansoura, Egypt; 4 OCMU, Faculty of medicine, Mansoura University, Mansoura, Egypt

**Keywords:** GISTs, Computed tomography, Radiomics, Risk stratification, Biomarkers, Gastroenterology, Oncology

## Abstract

To evaluate the feasibility of combining CT morphologic and radiomics features to predict malignancy risk in GIST patients and develop a multivariate regression model. Ninety-two patients with pathologically confirmed GISTs were enrolled. CT morphologic features were reviewed, and 42 radiomics features were extracted from the tumours on portal venous phase images. In the univariate analysis, each morphological and radiomics characteristic was compared between the low- and moderate/high-risk groups. Three multivariate regression models were performed for morphological, radiomics, and combined features to reveal the best predictor variables and model for malignancy risk prediction. In the multivariate regression model using CT morphologic features, the presence of tumour necrosis and tumour vessels were significant indicators for differentiating low-risk from moderate/high-risk GISTs (AUC = 0.81). In a model using CT radiomics features, skewness, total energy and GLCM_Idmn were significant indicators for differentiating both risk groups (AUC = 0.89). After combining both significant morphological and radiomic features in one model, the presence of tumour vessels and GLCM_Idmn were significant indicators for differentiating both risk groups (AUC = 0.90). Combined CT morphologic and radiomics analysis proved to be a useful method for differentiating low-risk from moderate/high-risk GISTs with high diagnostic performance. This integration has the power to guide comprehensive treatment strategies and determine the eligibility for adjuvant imatinib therapy. To the best of our knowledge, this is the first study to integrate CT morphological and radiomics features into a single predictive model for risk stratification of GISTs.

**Supplementary Information:**

The online version contains supplementary material available at 10.1038/s41598-026-66765-x.

## Introduction

Gastrointestinal stromal tumours (GISTs) are rare cancers accounting for 1–3% of all gastrointestinal neoplasms. However, they are considered the most common mesenchymal tumours of the gastrointestinal tract^1^.

GISTs are typically diagnosed in elderly individuals (approximately 60–65 years of age) with equal sex distributions and can also present in younger patients, such as those with Carney triad (childhood), neurofibromatosis type-1 (middle age), and familial GISTs (middle age)^2^. Approximately 10–30% of GISTs are malignant, but all such tumours have some malignant potential^3,4^. Therefore, a precise diagnosis of GISTs and differentiation from other tumours that share similar imaging findings is of utmost importance^5^.

Assessing the malignancy risk of GISTs is crucial as it significantly impacts treatment choices and prognosis. For low-risk cases, surgical removal is often the primary treatment. However, for high-risk cases, the tyrosine kinase inhibitor imatinib is recommended post-surgery. The follow-up plan is adjusted based on the risk assessment, with low-risk GISTs having a lower risk of relapse and consequently different follow-up requirements^6,7^.

The malignancy risk of GISTs is assessed based on tumour location, size and mitotic index, in accordance with the National Comprehensive Cancer Network (NCCN) guidelines^8^. The mitotic index is a crucial factor for malignancy risk assessment and has significant prognostic implications for GIST patients^3,9,10^. However, it can only be assessed through histological examination. Preoperative biopsy of suspected GISTs raises concerns due to the risk of bleeding and tumour cell seeding into the peritoneal cavity. Furthermore, a large-bore needle is typically required for core biopsy to ensure adequate sampling, as fine-needle aspiration may lead to insufficient tissue^11^.

Contrast-enhanced computed tomography (CE-CT) is the most widely used imaging technique in GIST diagnosis and staging because it can delineate the extent of the tumour and its metastasis. The characteristic CT features of GISTs typically include large, hypervascular, heterogeneous masses with necrosis, hemorrhage, or cystic changes seen, while smaller lesions may appear as homogeneous, endoluminal, or polypoid masses^12^.

Radiomics is an advanced texture analysis technique that quantifies tumour heterogeneity beyond visual assessment. This tool offers both qualitative and quantitative evaluations of tumour heterogeneity through analysing the distribution and relationship of pixel gray levels in the image^13–15^. The wider availability of free, open-source radiomics software programs has made CT radiomics analysis more accessible for researchers. Radiomics-based approaches are widely utilized in tumour staging, early diagnosis, differentiation, prognosis prediction, and treatment evaluation^16–20^. In addition, various studies have used radiomics with these software programs for risk stratification of GISTs^17,21–24^. High-grade tumours usually have higher cellularity and mitotic indices; moreover, malignant tumours frequently show greater vascularity and heterogeneous density than benign tumours do because of their greater incidence of internal haemorrhage and necrosis^13^.

This study aimed to evaluate the feasibility of combining CT morphologic and radiomics features to predict malignancy risk in GIST patients and develop a multivariate regression model. To the best of our knowledge, this is the first study to integrate CT morphological and radiomics features into a single predictive model for risk stratification of GISTs.

## Methods

### Patients

**Fig. 1:**
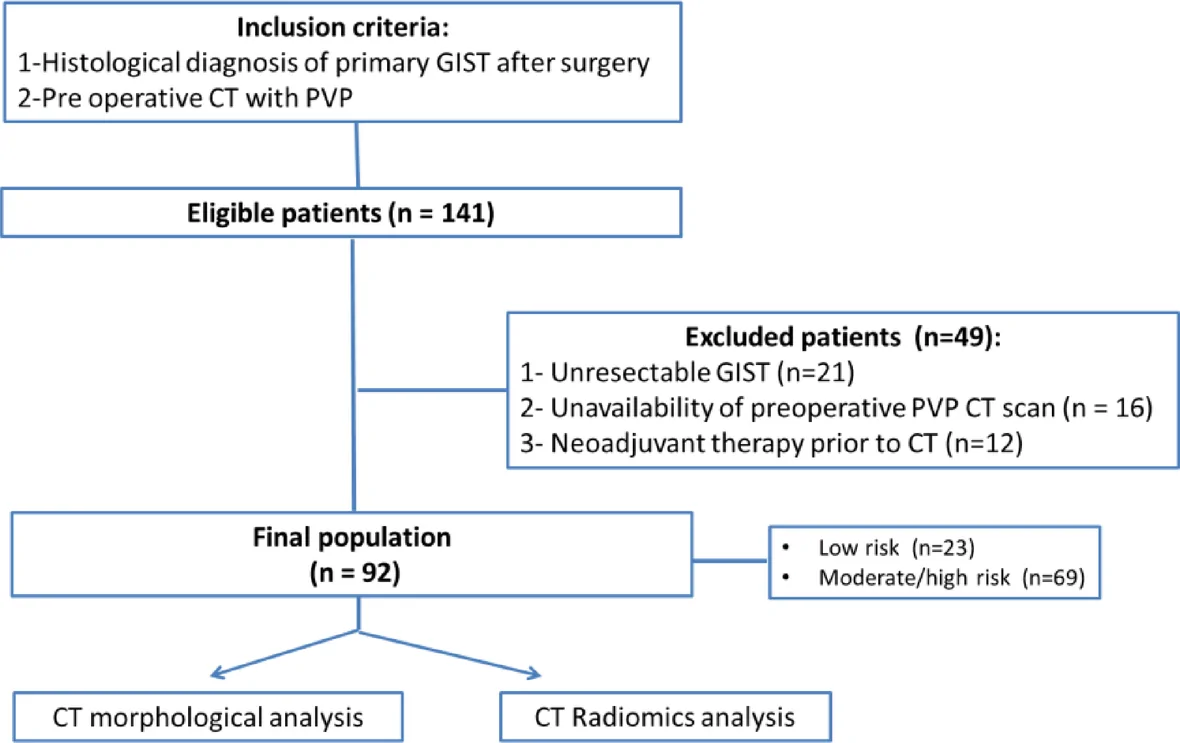
Flowchart of the study population. GIST: gastrointestinal stromal tumour; CT: computed tomography; PVP: portal venous phase.

The ethical approval for the study was obtained from Mansoura Faculty of Medicine Institutional Research Board (IRB Number: R.24.06.2686). The requirement for informed consent was waived by the same Institutional Research Board due to the retrospective design of the study. This study was conducted in accordance with the Declaration of Helsinki and adhered to relevant institutional and national guidelines and regulations. Patients were enrolled if they had a pathologically confirmed primary GIST from surgically resected specimens and CE-CT of the abdomen in the portal-venous phase (PVP) before treatment without prior exposure to neoadjuvant therapy. Between January 2019 and December 2023, a pathology database search at a single institution revealed 141 patients with a pathological diagnosis of primary GIST. Twenty-one patients with unresectable tumours were excluded because the risk of malignancy of GISTs is indiscernible according to biopsy samples. Sixteen patients were excluded because PVP CT examination was unavailable. Twelve patients with a history of neoadjuvant therapy prior to CT were also excluded. Hence, the final cohort included 92 patients (45 men [49%], 47 women [51%]), with a mean age of 57 ± 13 (SD) years. They were categorized into the low-risk group (*n* = 23; very low/low- risk cases) and the high-risk group (*n* = 69; intermediate-risk cases: 16, high-risk cases; 53)^17,25–27^(Fig. 1). Patient demographic, clinical and treatment data were retrieved from medical records. Patients had undergone CE-CT examinations for the following symptoms/findings: abdominal pain (*n* = 67); hematemesis (*n* = 6); an incidentally discovered lesion during ultrasonography for unrelated conditions (*n* = 5); melena/bleeding per rectum (*n* = 7); abdominal enlargement (*n* = 2); dysphagia (*n* = 2); jaundice (*n* = 1); anaemia (*n* = 1); and weight loss (*n* = 1). The interval between surgery and postcontrast CT examination was approximately 52 ± 84 (SD) days. The malignant risk of GISTs in all patients was evaluated by a specialized pathologist who used the tumour location, size, and mitotic index for risk assessment according to the commonly used risk stratification system proposed by the Armed Forces Institute of Pathology (AFIP)^8,28^.

### CT acquisition technique

Triphasic CT was performed in 39/92 (42.4%) patients, whereas CE-CT with PVP was obtained in 53/92 (57.6%) patients. Different CT scanners were used, including GE Healthcare (Revolution EVO, Bright speed, and Optima CT520 series, Waukesha, WI, USA), Toshiba Medical Systems (Aquilion prime 128 detectors, Otawara, Japan), and Philips Healthcare (Brilliance 64, Amsterdam, the Netherlands). The imaging parameters were as follows: a tube voltage of 100–140 kV, 149–500 mAs, a pitch of 0.8–1.7, a field of view of 357–500 mm, a matrix of 512 × 512, a 2–3 mm slice thickness and a 0.8–5 mm interslice gap. Axial, sagittal, and multiplanar reconstructed images with thicknesses of 2–5 mm were acquired. For triphasic CT, noncontrast abdominal acquisition was initially performed, followed by arterial and portal venous phase acquisition 21–28 and 55–65 s after intravenous administration of an adjusted dose of iodinated contrast agent (2 mL/kg, 350 ng/mL) at a rate of 3–5 mL/sec. For CE-CT, PVP images were obtained 70 s after intravenous administration of contrast agent.

### CT morphological analysis

All patients’ CT images were scrutinized by two radiologists, with 12 and 10 years of experience in diagnostic imaging, respectively, who were blinded to the pathologic risk of GISTs. For morphological assessment, the following CT features were assessed: primary tumour location (gastric, small intestinal, peritoneal, etc.), size (maximum longitudinal axis and shot axis were measured three times by one reviewer on the transverse plane then averaged), margins (well-defined or ill-defined), shape (regular if round or ovoid and irregular if lobulated), growth pattern (endoluminal, exophytic, or mixed)^29^, central or peripheral feeding vessels (tumour vessels), necrosis (absent, mild if < 50 of the tumour, and severe if > 50% of the tumour), enhancement pattern (homogeneous or heterogeneous), and enhancement degree (mild if enhancement degree is ≤ muscle, moderate if > muscle, but ≤ liver, and marked if > liver). Tumour necrosis, enhancement pattern, and enhancement degree were evaluated during PVP. The presence of fistulization between the tumour and the adjacent bowel lumen (indicated by the presence of air inside the lesion) was also assessed. In addition, the presence of calcification, ascites, direct organ invasion, and regional lymphadenopathy (if the nodal short axis diameter was > 1 cm) were determined.

### CT radiomics analysis

**Fig. 2:**
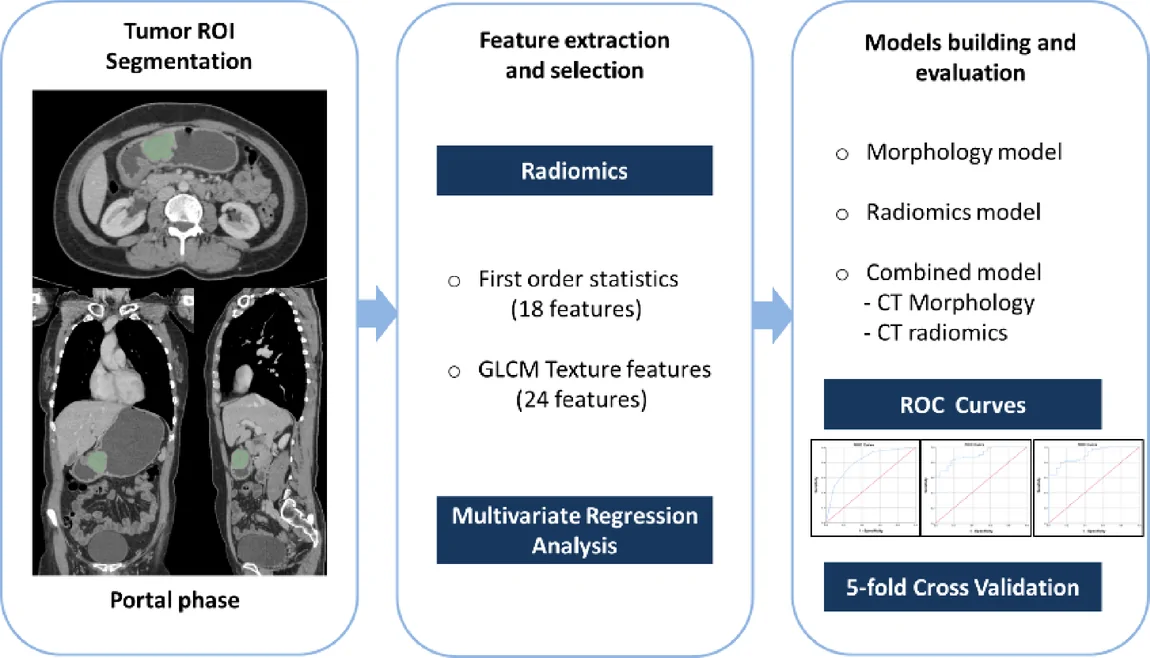
Flowchart of tumour ROI segmentation, radiomics extraction, models building and evaluation.

For tumour segmentation and radiomics feature extraction, free open source 3D Slicer software (version 5.5.0 built 2023.10.10; https://www.slicer.org/) was applied^30^. This process was performed by a well-trained radiologist, with 14 years of experience in abdominal imaging, who was not informed about the pathological risk of the tumour. First, the PVP CT images were loaded onto the 3D slicer platform, and abdominal soft tissue window settings were adjusted (W: 400, L: 50). On each axial section, a volumetric ROI was manually drawn largest possible to include the whole lesion Necrosis within the tumour was included, precisely excluding adjacent calcification, air or water in the digestive tract^17,31,32^(Fig. 2). We standardized CT images from various scanners by resampling to a uniform voxel size of 2 × 2 × 2 mm³ and normalizing with a window width of 400 and window level of 50 before extracting features. Finally, a radiomics extension (PyRadiomics library)^33^ was used to extract radiomics features on the basis of the Imaging Biomarker Standardization Initiative (IBSI) definitions^34^. Among the different radiomics features, a total of forty-two features for each tumour were extracted, including 18 features from the first-order statistics and 24 features from the gray-level co-occurrence matrix (GLCM). The first-order statistics measure the distribution of CT attenuation numbers or voxel values, such as the mean, standard deviation (SD), median, minimum, maximum, entropy, skewness and kurtosis of the histogram. The GLCM is a second-order statistical method that provides measures of the heterogeneity inside the lesion through assessing spatial relationships between adjacent voxels^31,35,36^.

### Statistical analysis

The Statistical Package of Social Science (SPSS) program (Standard version 24) for Windows was operated for data analysis. First, data normality was assessed using the one-sample Kolmogorov-Smirnov test. Normally distributed variables are presented as mean ± SDs, while non-normally distributed variables are expressed as median (range). Group comparisons utilized independent t-test for normally distributed data and Mann‒Whitney U test for non-normally distributed data. Qualitative data are presented as numbers and percentages. The chi-square test was used to test associations between categorical variables, whereas Fisher’s exact test was used when the expected cell count was < 5.

Interobserver agreement was calculated by using kappa coefficients, where almost perfect agreement was achieved, with a kappa value of 0.81–1.00; substantial agreement, with 0.61–0.80; moderate agreement, with 0.41–0.60; fair agreement, with 0.21–0.40; and slight agreement, with 0.00–0.20^37^.

To differentiate low-risk GISTs from moderate/high-risk GISTs, three multivariate logistic regression models were constructed using qualitative morphologic features in the first one, quantitative radiomics features in the second one, and a combination of both features in the third one. Only significant variables in the univariate analysis were included in the logistic regression model via the forward stepwise selection technique to predict the most significant determinants and to control for possible interactions and confounding effects. We excluded tumour site and size from univariate analysis since they’re already established risk factors. This allowed us to explore other predictive features that could complement these factors in a clinical setting. Furthermore, receiver operating characteristic (ROC) curves were obtained for each model. *P* < 0.05 was considered to indicate a significant difference. Difference between the areas under two independent ROC curves was assessed using DeLong’s test. Finally, the internal validity of the combined model was assessed using cross validation technique applying a 5-fold approach with cross tabulation between actual and predicted values to assess accuracy, precision, recall and F1 score.

## Results

### CT morphological analysis

**Fig. 3:**
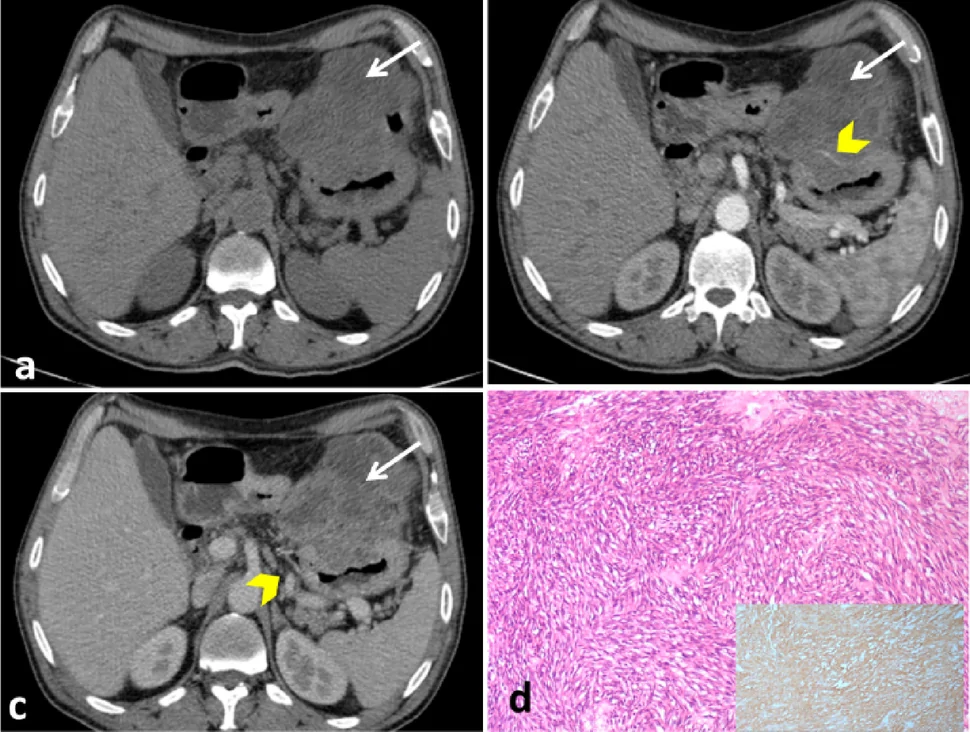
High-risk gastric GIST in a patient presenting with abdominal pain. Axial non-enhanced CT image (**a**) shows an irregular hypodense gastric mass with a mixed growth pattern (arrow). Arterial (**b**) and portal venous phase (PVP) contrast-enhanced CT images (**c**) show a mass with a mixed growth pattern, heterogeneous enhancement, marked necrosis (arrows) and tumour vessels (arrowheads). It measures 15 × 12 cm in maximum dimensions. Radiomics first order maximum = 119, minimum= -12, skewness= -0.19, and total energy = 1,778,713,488. (**d**) Microscopic examination (200x) revealed intersecting fascicles of spindle-shaped tumour cells with increased cellularity, moderate atypia and an increased mitotic index (more than 5/50 HPF). Tumour cells were diffusely positive for DOG-1 (inset, 200x), which is consistent with high-risk GIST.

**Fig. 4:**
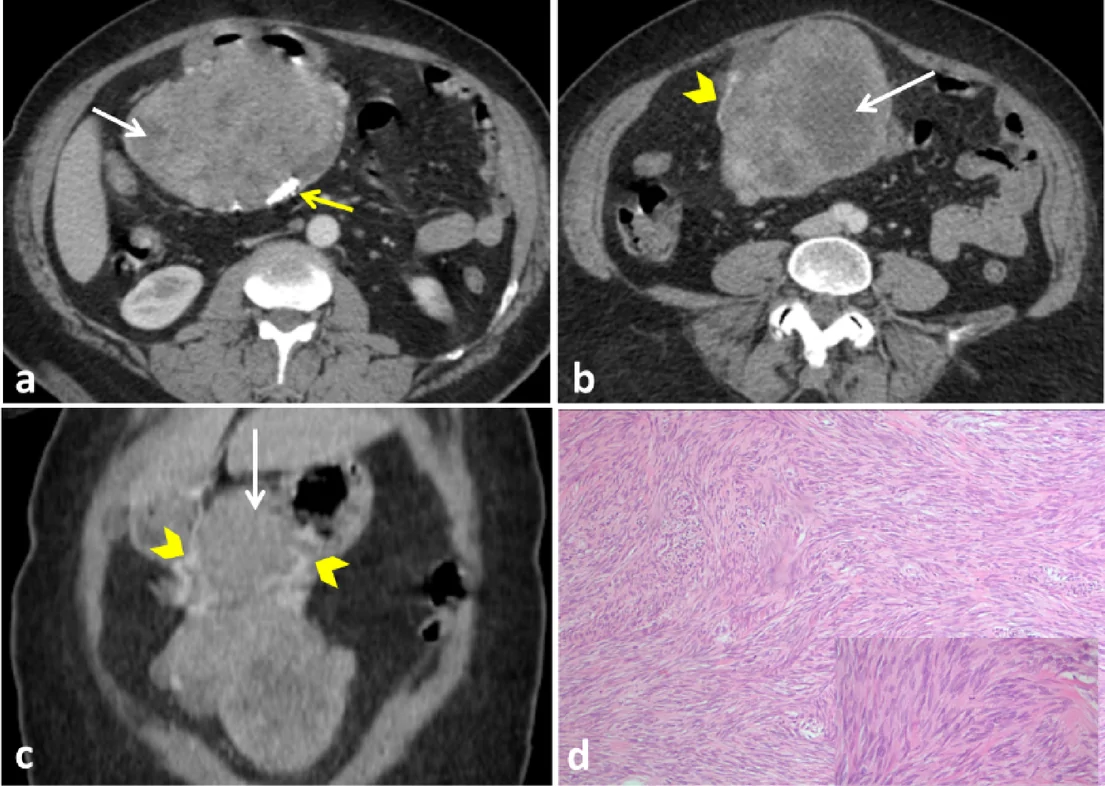
High-risk gastric GIST in a patient complaining of abdominal pain. (**a**, **b**) Axial portal venous phase (PVP) contrast-enhanced CT images at the upper (**a**) and lower (**b**) levels show an irregular heterogeneously enhancing gastric mass with peripheral coarse calcifications (yellow arrow), marked necrosis (white arrows), and tumour vessels at the periphery (arrowhead). (**c**) Coronal PVP contrast-enhanced CT images showing a mixed growth pattern (white arrow) and tumour vessels (arrow heads). It measures 13 × 9 cm in maximum dimensions. Radiomics first order maximum = 1539, minimum= -678, skewness= -2.3, total energy = 1,934,389,216. (**d**) Microscopic examination revealed intersecting fascicles of spindle-shaped tumour cells with increased cellularity, moderate atypia (200x) and an increased mitotic index (more than 5/50 HPF) (inset, 400x), which is consistent with high-risk GIST.

**Fig. 5:**
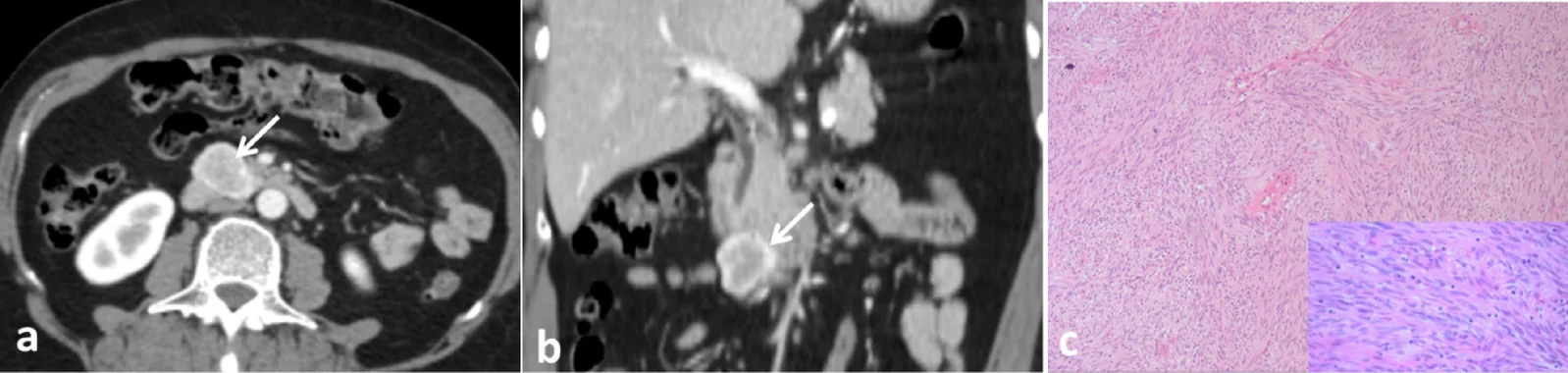
Low-risk duodenal GIST in a patient complaining of abdominal pain. (**a**, **b**) Axial and coronal portal venous phase (PVP) contrast-enhanced CT images showing a well-defined regular heterogeneous enhancing mass arising from the 3rd part of the duodenum with a mixed growth pattern (arrows). Neither necrosis nor tumour vessels could be observed within the mass. It measures 3 × 2 cm in maximum dimensions. Radiomics first order maximum = 140, minimum= -18, skewness = 0.12, total energy = 4,847,732,968. (**c**) Microscopic examination revealed intersecting fascicles of spindle-shaped tumour cells with mild atypia (200x), and a low mitotic index (less than 5/50 HPF) (inset, 400x), which is consistent with low-risk GIST.

Gastric location was more prevalent in low-risk patients (78.3%) than in moderate/high-risk patients (36.2%) (*p* = 0.003), whereas maximum tumour size was more prevalent in moderate/high-risk patients than in low-risk patients (*p* = 0.000). Ill-defined margins and irregular shapes were more common in moderate/high-risk patients (49.3% and 89.9%) than in low-risk patients (13.0% and 65.2%, respectively). Additionally, moderate/high-risk patients more frequently had necrosis (*p* = 0.00), heterogeneous patterns of enhancement (*p* = 0.0007), and tumour vessels (*p* = 0.001) than low-risk patients did. Tumour fistulization and direct organ invasion were observed only in moderate/high-risk patients (18.8% and 21.7%, respectively). In addition, there was a significant difference in growth pattern between the two risk groups (*p* = 0.004). However, the other qualitative variables were not significantly different between the two groups (Table 1) (Figs. 3, 4 and 5).

**Table 1:** Patient demographics and CT morphologic features.

Variable	Total (no = 92)	Risk stratification		Test of significance
		Low (*n* = 23)	Moderate/High (*n* = 69)	
Sex Male Female	45(48.9%) 47(51.1%)	11(47.8%) 12(52.2%)	34(49.3%) 35(50.7%)	FET *p* = 1
Age Median (Min–Max)	58(12–83)	61(12–76)	57(26–38)	Z = 0.853 *p* = 0.394
Size LA (mm) Mean ± SD	100.35 ± 59.137	47.13 ± 20.526	118.09 ± 57.154	t = 8.757 *p* = 0.000*
Size SA (mm) Mean ± SD	74.61 ± 42.988	37.57 ± 16.640	86.96 ± 42.006	t = 8.054 *p*=0.000*
Site Stomach Small intestine Peritoneal Others	43(46.7%) 30(32.6%) 9(9.8%) 10(10.9%)	18(78.3%) 5(21.7%) 0 0	25(36.2%) 25(36.2%) 9(13%) 10(14.5%)	χ2 = 13.964 *p* = 0.003*
Tumour margin Well-defined Ill- defined	55(59.8%) 37(40.2%)	20(87.0%) 3(13.0%)	35(50.7%) 34(49.3%)	FET *p* = 0.03
Tumour shape Regular Irregular	15(16.3%) 77(83.7%)	8(34.8%) 15(65.2%)	7(10.1%) 62(89.9%)	FET *p* = 0.01
Necrosis Absent < 50% > 50%	19(20.7%) 27(29.3%) 46(50%)	12(52.2%) 7(30.4%) 4(17.4%)	7(10.1%) 20(29.0%) 42(60.9%)	χ2 = 21.288 *p* = 0.00*
Calcification Present Absent	14(15.2%) 78(84.8%)	1(4.3%) 22(95.7%)	13(18.8%) 56(81.2%)	FET *p* = 0.176
Growth patterns Endoluminal Exophytic Mixed	8(8.7%) 52(56.5%) 32(34.8%)	5(21.7%) 7(30.4%) 11(47.8%)	3(4.3%) 45(65.2%) 21(30.4%)	χ2 = 11.192 *p* = 0.004*
Enhancement pattern Homogenous Heterogeneous	14(15.2%) 78(84.8%)	10(43.5%) 13(56.5%)	4(5.8%) 64(92.8%)	χ2 = 19.134 *p* = 0.00*
Enhancement degree Mild Moderate Marked	8 (8.7%) 63(68.5%) 21(22.8%)	4(17.4%) 15(65.2%) 4(17.4%)	4(5.8%) 48(69.6%) 17(24.6%)	χ2 = 3.111 *p* = 0.21
Presence of intramural vessels Present Absent	50(54.3%) 42(45.7%)	5(21.7%) 18(78.3%)	45(65.2%) 24(34.8%)	FET *p* = 0.001*
Direct organ invasion Present Absent	15(16.3%) 77(83.7%)	0(0%) 23(100%)	15(21.7%) 54(78.3%)	FET *p* = 0.018*
Fistulation Present Absent	13(14.1%) 79(85.9%)	0(0%) 23(100%)	13(18.8%) 56(81.2%)	FET *p* = 0.033*
Metastasis No Peritoneal Hepatic Both	75(81.5%) 7(7.6%) 4(4.3%) 6(6.5%)	23(100%) 0(0%) 0(0%) 0(0%)	52(75.4%) 7(10.1%) 4(5.8%) 6(8.7%)	χ2 = 6.951 *p* = 0.073
Ascites Present Absent	6(6.5%) 86(93.5%)	0(0%) 23(100%)	6(8.7%) 63(91.3%)	FET *p* = 0331
Regional lymphadenopathy Present Absent	7(7.6%) 85(92.4%)	1(4.3%) 22(95.7%)	6(8.7%) 63(91.3%)	FET *p* = 0.657

* Significant difference between the low-risk and moderate/high-risk groups.

There was almost perfect agreement between the observers regarding the presence of tumour necrosis (k = 0.86), calcification (k = 0.96), fistulization (k = 1), and metastasis (k = 1). There was substantial agreement for the presence of ascites (k = 0.73) and regional lymphadenopathy (k = 0.78). Moderate agreement was achieved for the tumour margin (k = 0.59), tumour shape (k = 0.58), growth pattern (k = 0.60), enhancement pattern (k = 0.52), enhancement degree (k = 0.58), presence of tumour vessels (k = 0.59), and direct organ invasion (k = 0.59). The detailed results are shown in Supplementary table S1.

### CT radiomics analysis

Fourteen first-order features and only two GLCM features were significantly different between low-risk and moderate/high-risk patients with GISTs. The first-order 10th percentile, mean, median, minimum, and root mean squared values were significantly greater for low-risk GISTs (*p* = 0.001, *p* = 0.010, *p* = 0.009, *p* = 0.004, *p*=0.026, respectively) than for moderate/high-risk GISTs, whereas the other features were significantly greater for moderate/high-risk GISTs. The detailed results are shown in Supplementary table S2.

### Univariate and multivariate logistic regression models

In the univariate analysis of CT morphologic features, significant differences in the tumour margin, shape, necrosis, enhancement pattern, and tumour vessels were observed between low-risk patients and moderate/high-risk patients. Whilst, univariate analysis of the CT radiomics features revealed significant differences in 14 of the 42 features between low-risk and moderate/high-risk GISTs, all of which were first-order features (*n* = 13), with the exception of one second order feature feature, gray-level co-occurrence matrix – inverse difference moment normalized (GLCM_Idmn) (Tables 2 and 3).

**Table 2:** Multivariate regression model using CT morphologic features.

Morphologic features	Univariate regression		Multivariate regression	
	*P* value	OR (95%) CI	*P* value	OR (95%) CI
Tumour margin Well-defined (r) Ill- defined	0.005	6.476(1.761–23.812)		
Tumour shape Regular (r) Irregular	0.009	0.212(0.066–0.676)		
Necrosis Absent < 50% > 50% (r)	0.000	4.270(2.123–8.589)	0.002	3.288(1.559–6.934)
Enhancement pattern Homogenous (r) Heterogeneous	0.000	12.414 (3.387–45.493)		
Tumour vessels Present Absent (r)	0.001	6.750 (2.230-20.436)	0.046	3.456(1.024–11.666)

(r): reference group, CI: confidence interval.

* Significant difference between the low-risk and moderate/high-risk groups.

**Table 3:** Multivariate regression model using CT radiomics features.

Texture features	Univariate regression		Multivariate regression	
	*P* value	OR (95%) CI	*P* value	OR (95%) CI
10 Percentile	0.002	0.968(0.949–0.988)		
Energy	0.002	1.000(1.000–1.000)		
Interquartile Range	0.009	1.116(1.027–1.213)	**-**	**-**
Maximum	0.031	1.019(1.002–1.036)		
Mean Absolute Deviation	0.011	1.209(1.043-1.400)		
Mean	0.018	0.978(0.959–0.996)		
Median	0.014	0.977(0.959–0.995)		
Minimum	0.011	0.982(0.968–0.996)		
Range	0.001	1.024(1.010–1.039)		
Robust Mean Absolute Deviation	0.009	1.310(1.070–1.603)		
Skewness	0.011	6.592(1.530-28.394)	0.083	6.364 (0.786–51.545)
Total Energy	0.002	1.000(1.000–1.000)	0.041	1.000 (1.000–1.000)
Variance	0.033	1.003(1.000-1.006)		
GLCM_ Idmn	0.037	0.848(0.793 − 0.075)	0.063	0.013 (0.000-1.265)

CI: confidence interval.

* Significant difference between the low-risk and moderate/high-risk groups.

**Fig. 6:**
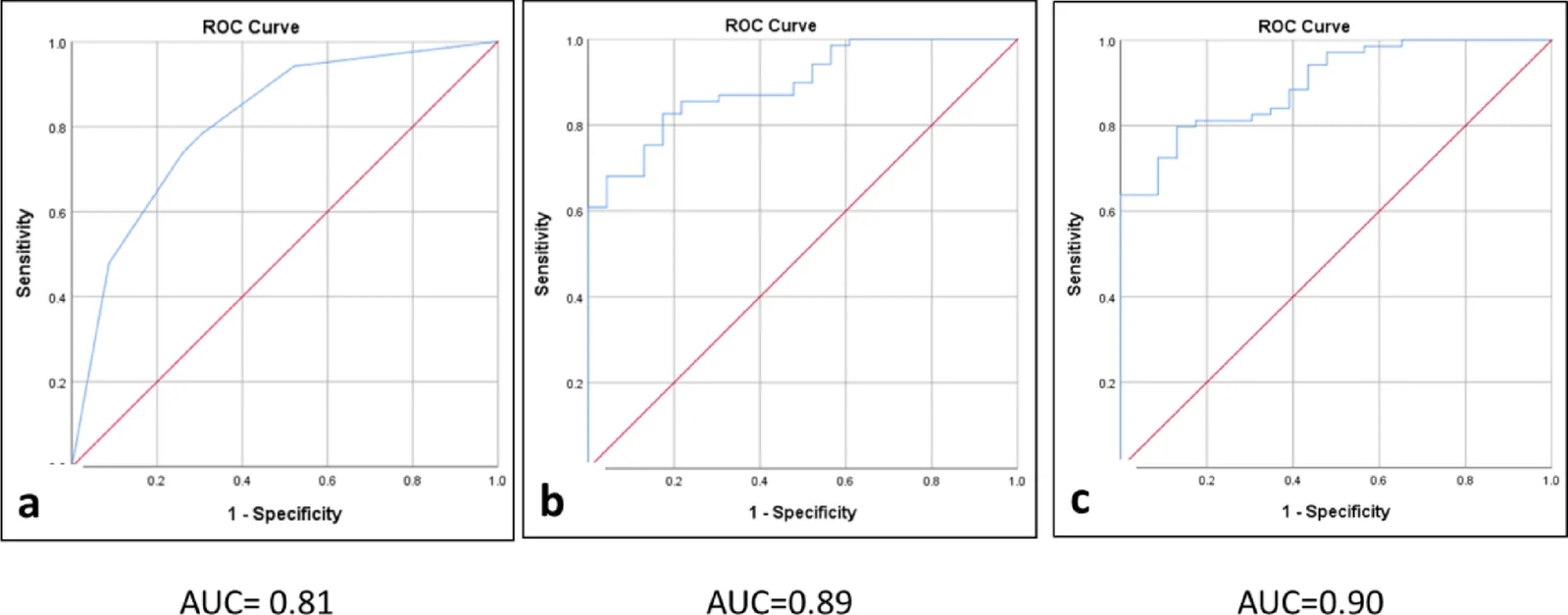
Graphical illustrations of the receiver operating characteristic (ROC) curves of the three multivariate logistic regression models. (**a**)The morphological model, (**b**) the radiomics model, and (C) the combined model. AUC: area under the curve.

In the multivariate logistic regression model using CT morphologic features, the presence of tumour necrosis and tumour vessels were significant indicators for differentiating low-risk from moderate/high-risk GISTs, and the AUC was 0.81 (Table 2). In the multivariate logistic regression model using CT radiomics features, two first-order features (skewness and total energy) and one GLCM feature (GLCM_Idmn) were significant indicators for differentiating both risk groups, and the AUC was 0.89 (Table 3). After combining both significant morphological and radiomics features in one logistic regression model, the presence of tumour vessels and GLCM_Idmn were significant indicators for differentiating both risk groups, with an AUC = 0.90 (Table 4). The combined model showed higher AUC (0.90), sensitivity (81.2%), and specificity (78.3%) than the morphological model. However, there was no significant difference between the AUC of both models (*P* = 0.186) as shown in Fig. 6; Table 5. Moreover, 5-fold cross validation was done to assess internal validity of the combined model and demonstrated that the results were valid across different folds as shown in Supplementary tables S3.

**Table 4:** Multivariate regression model using combined CT morphological and radiomic features.

	*P* value	Odds ratio	95% CI
GLCM_ Idmn	0.010^*^	0.725	0.612–0.834
Tumour vessels	0.015^*^	0.136	0.027–0.677

* Significant difference between the low-risk and moderate/high-risk groups.

**Table 5:** Delong method for assessment of difference between different ROC curves.

	Morphology model	Radiomics model	Combined model
Area under curve	0.812	0.894	0.90
95%CI	0.707–0.917	0.829–0.959	0.833–0.961
Standard error	0.054	0.03	0.03
Sensitivity %	78.3	87	81.2
Specificity %	69.6	69.6	78.3
TN	16	16	18
TP	54	60	56
Z	Z1 = 1.26 Z2 = 1.32 Z3 = 0.058		
P value	P1 = 0.205 P2 = 0.186 P3 = 0.476		

Z1, P1: difference between ROC curve of morphological model and radiomics model. Z2, P2: difference between ROC curve of morphological model and combined mode, Z3, P3: difference between ROC curve of radiomics.

## Discussion

Risk stratification for primary GISTs is pivotal owing to the emergence of adjuvant systemic treatments, especially for high-risk tumours, to ensure a better prognosis and prolonged survival^6^. This stratification is determined by the location, size, and mitotic index of the tumour. Among these parameters, the mitosis index can be confirmed only pathologically. If a high mitotic index is present, the proliferation of the tissues is rapid, causing increased blood supply to the tumour and complications such as bleeding, necrosis, and cystic changes. Therefore, the more frequent heterogeneous enhancement pattern, necrosis, and tumour vessels in moderate/high-risk patients in the present study may be positively correlated with higher mitotic counts, making our observations reasonable. Previous studies have described large tumours (> 11 cm), necrosis, heterogeneous density, and hepatic or peritoneal metastasis on CT images as findings suggesting high-grade GIST or a high mitotic index of the tumour^29,38–40^.

Moreover, our multivariate logistic regression model using CT morphologic features revealed that the presence of tumour necrosis and tumour vessels were significant indicators for differentiating low-risk from moderate/high-risk GISTs (AUC = 0.81). In a previous retrospective study^29^, a multinomial regression model revealed that only tumour size, pattern of growth, and tumour vessels were independent predictors for risk stratifications. In another series, a logistic regression model was built that showed only lesion size (> 5 cm) and exophytic/mixed growth pattern as independent predictors for high-risk GISTs^40^. However, in our study, we built a regression model after excluding tumour size and location from the analysis to avoid bias in selection, as both variables are well known to be risk determinants, and we aimed to find imaging biomarkers homologous to the mitotic index.

Radiomics has recently emerged as a promising tool for quantifying lesion heterogeneity and extracting quantitative data from radiological images that are imperceptible to the naked eye. This capability is crucial; as such features are known to be key determinants of tumour behavior and resistance to therapy^14–16^.

In the current study, the first-order maximum was significantly greater, whereas the minimum and mean were significantly lower in moderate/high-risk patients with GISTs. We speculate that the first-order maximum, representing the maximum CT value, probably reflecting the most ample blood supply within the tumour on CE-CT images, whereas minimum represents the minimum CT value, signifying necrosis, cystic degeneration, or even the poorest blood supply within the tumour on CE-CT images. These results align with previous findings that reported corresponding correlations between maximum and minimum CT value correlations^17^. However, no significant differences were observed in the mean values between the two risk groups with varying malignancy risk. A prior studyreported lower mean values in moderate/high-risk GISTs compared to our results^22^; but their analysis was limited to CT radiomics and morphologic features, without integrating both as we did.

A previous study has reported that entropy based on PVP images significantly varies and correlates with different malignancy risks of GISTs^17^. Additionally, another study found that entropy differed significantly between high-grade and low-grade urothelial carcinomas^41^. This parameter reflects the heterogeneous enhancement of tumours caused by necrosis, cell proliferation and a disordered blood supply. However, in the present study, entropy did not significantly differ between the two risk groups, yielding findings similar to those of another study^22^. This discrepancy in results could be attributed to the heterogeneity of CT scanners, different methods of analysis, and various tumour types. Further studies are warranted to clarify the importance of these different parameters.

Additionally, moderate/high-risk GISTs were characterized by significantly higher skewness values. Our findings are in accordance with previous research^22^. However, kurtosis was greater in high-risk than in low-risk patients in that research, unlike our findings, where no significant difference was noted in kurtosis measurements between the risk groups. This could be due to the greater number of low-risk GISTs in their study (63%) than in our study (25%), which could affect the fraction of heterogeneous tumours with higher kurtosis values.

In the present study, two second-order parameters (GLCM Idmn and GLCM Idn) were significantly greater in moderate/high-risk patients than in high-risk patients, which were in accordance with the results of a previous study that reported similar significant parameters between neuroblastoma and sinonasal squamous cell carcinoma^36^. We selected the GLCM among several reported second-order statistical methods, as it was the most frequently used feature in several previous studies^35,42–45^. A prior study leveraging MRI radiomics successfully predicted prostate cancer bone metastasis preoperatively, revealing significant correlations between GLCM texture parameters and primary tumour biology^44^. Using MRI texture analysis by GLCM features (contrast and homogeneity) to predict poorly differentiated head and neck squamous cell carcinoma, a previous study revealed that the relative contrast was significantly lower in the poorly differentiated carcinoma than the well/moderately differentiated one, while the homogeneity was higher in poorly differentiated tumour^45^.

A multivariate logistic regression model using quantitative radiomics features revealed that first-order skewness and total energy as well as second-order GLCM_Idmn were significant indicators for differentiating both risk groups with nearly similar performance to the morphological model (AUC of 0.89). After integrating both significant morphological and radiomics features in one logistic regression model, the presence of tumour vessels and GLCM_Idmn were found to be the most significant indicators for differentiating both risk groups with the highest AUC (= 0.90) compared with the other two models. Despite non-significant AUC differences among the three models, the combined model demonstrated a more favorable balance of sensitivity and specificity, which may enhance its utility in clinical decision-making. GLCM_Idmn reflects local image homogeneity; its significance suggests that tumour uniformity at a voxel level correlates with malignancy potential. In addition, the significant role of the GLCM in our findings was highlighted. One previous study revealed that the GLCM could be superior to first-order statistics in detecting image heterogeneity^45^.

Second-order and higher-order radiomics features use adjacent gray-level difference matrices to analyse texture in a specific direction. Accordingly, they can assess the location and relationships between three or more pixels, alongside the first-order parameters provided by the 3D slicer program. These parameters are expected to represent more delicate or complex configurations of tissue textures and have shown promising results in several recent studies^32,36,44^. In this situation, the conveniently available open-source programs can facilitate an objective evaluation of the usefulness of each parameter and validate their use as imaging biomarkers for various tumours.

Our study was restricted to the portal venous phase of contrast-enhanced CT, and it is worth situating this predictive performance against other functional and molecular imaging modalities that have been explored for GIST risk stratification. PET/CT-based SUVmax correlates well with Ki-67 and mitotic count in GIST, and in gastric GIST a SUVmax cutoff of 2.60 reached an AUC of 0.967 (sensitivity 100%, specificity 83.3%) for malignancy. This nominally outperforms CT radiomics (~ 0.73–0.90), but PET/CT studies tend to be small, single-center, and limited to FDG-avid tumors, inflating accuracy, and PET/CT remains costlier, less accessible, and at least as radiation-intensive as multiphase CT^46^. Similarly, a prospective study comparing conventional CT, CT perfusion, and MR-DWI/IVIM in gastric GIST found lower ADC values independently predicted high-risk disease, with a combined morphologic-functional model reaching an AUC of 0.869. Yet, ADC alone tends to underperform relative to radiomics-morphologic CT models, since it captures a single scalar value rather than rich textural information, and is more prone to motion artifact and b-value variability in the abdomen^47^.

This study had several limitations that should be highlighted. First, our study is limited by its retrospective nature. The sample size was relatively small, limiting the ability to extrapolate the results and ensure strong model robustness. Nevertheless, this single-centre experience provides valuable insights within a condensed timeframe, complementing existing literature. Second, our predictive models were not validated on an external dataset due to limited sample size. Instead, we employed 5-fold cross-validation to evaluate internal validity and assess predictive consistency of our combined model. Although our results appear promising, a prospective validation study in a large cohort of patients is warranted to establish the potential role of CT texture features in combination with morphologic features as prognostic biomarkers in patients with GISTs. Additionally, the retrospective design necessitated utilizing images from various CT scanners, potentially introducing variability. This heterogeneity in imaging protocols may impact data consistency and result reliability, underscoring the need for prospective investigations with standardized protocols and larger cohorts. Moreover, the radiomics analysis was limited to 42 first-order and GLCM features, excluding the Shape, GLRLM, GLSZM, GLDM, and NGTDM features available in open-source platforms like PyRadiomics. We restricted our selection to first-order and GLCM features because they are the most commonly utilized in previous GIST risk stratification research and have shown the best comparative results. Furthermore, shape features were excluded since they were already accounted for in the morphological analysis. Finally, tumour ROIs manual delineation process relied heavily on radiologists’ experience, requiring significant time and effort. Future research should focus on deep learning-based automated segmentation tools to enhance efficiency and reduce inter-operator variability.

## Conclusions

Combined CT morphologic and radiomics analysis has proven to be a useful method for differentiating low-risk from moderate/high-risk GISTs with high diagnostic performance. This combined imaging approach has the potential to serve as a virtual biopsy, aiding treatment planning and personalized surveillance by predicting GIST mitotic index without invasive procedures.

## Supplementary Information

Below is the link to the electronic supplementary material.

Supplementary Material 1

## Data Availability

Due to ethical concerns and participant privacy, the datasets supporting the findings of this study are not publicly available but can be obtained from the corresponding author upon reasonable request, subject to institutional review board approval.

## Abbreviations

GISTs: Gastrointestinal stromal tumours
NCCN: National comprehensive cancer network
CE CT: Contrast-enhanced computed tomography
SD: Standard deviation
ROI: Region of interest
PVP: Portal-venous phase
AFIP: Armed forces institute of pathology
GLCM: Gray-level co-occurrence matrix
GLCM-Idmn: Gray-level co-occurrence matrix – inverse difference moment normalized
IBSI: Imaging biomarker standardization initiative
AUC: Area under curve
