# Supplementary material for: Integrating CT radiomics and morphologic features for preoperative risk stratification of gastrointestinal stromal tumours
Source: Sci Rep. 2026 Aug 25;16:26578. doi: 10.1038/s41598-026-66765-x (PMC13503734; doi:10.1038/s41598-026-66765-x)
Supplement: Supplementary file 1 [file 41598_2026_66765_MOESM1_ESM.docx]

**Supplementary table S1: Inter-observer agreement of CT morphologic features**

| Characteristics | Observer 1 | Observer 2 | Kappa agreement % |
| --- | --- | --- | --- |
| Tumor margin  Well-defined  Ill- defined | 55(59.8%)  37(40.2%) | 57(62%)  35(38%) | 58.9% |
| Tumor shape  Regular  Irregular | 15 (16.3%)  77 (83.7%) | 27(29.3%)  65(70.7%) | 57.7% |
| Necrosis  Absent  <50%  >50% | 19(20.7%)  27(29.3%)  46(50%) | 23(25%)  27(29.3)  42(45.7%) | 86.3% |
| Calcification  Present  Absent | 15(16.3%)  77(83.7%) | 14(15.2%)  78(84.8%) | 95.9% |
| Growth patterns  Endoluminal  Exophytic  Mixed | 8(8.7%)  52(56.5%)  32(34.8%) | 10(10.9%)  57(62%)  25(27.2%) | 60.2% |
| Enhancement pattern  Homogenous  Heterogeneous | 14(15.2%)  78(84.8%) | 10 (10.9%)  82 (89.1%) | 52.3% |
| Enhancement degree  Mild  Moderate  Marked | 8(8.7%)  63(68.5%)  21(22.8%) | 8(8.7%)  53(57.6%)  31(33.7%) | 58.3% |
| Direct organ invasion  Present  Absent | 15(16.3%)  77(83.7%) | 10(10.9%)  82 (89.1%) | 58.6% |
| Fistulation  Present  Absent | 13(14.1%)  79(85.9%) | 13(14.1%)  79(85.9%) | 100% |
| Metastasis  No  Peritoneal  Hepatic  Both | 75(81.5%)  7(7.6%)  4(4.3%)  6(6.5%) | 75(81.5%)  7(7.6%)  4(4.3%)  6(6.5%) | 100% |
| Presence of intramural vessels  Present  Absent | 50 (54.3%)  42 (45.7%) | 47(51.1%)  45(48.9%) | 58.6% |
| LN  Present  Absent | 7 (7.6%)  85(92.4%) | 8(8.7%)  84(91.3%) | 78.2% |
| Ascites  Present  Absent | 6(6.5%)  86(93.5%) | 10(10.9%)  82 (89.1%) | 72.8% |

**Supplementary table S2: CT radiomics features**

|  | Total  (no=92) | | Risk | | Test of significance |
| --- | --- | --- | --- | --- | --- |
|  |  |  | **Low** | **Moderate/high** |  |
| First order 10 Percentile  Median (Min-Max) | | 24 (-46-108) | 37(-1-106) | 18(-46-108) | Z= 3.36  p= 0.001 ⃰ |
| First order 90 Percentile  Median (Min-Max) | | 76 (36-205) | 84 (47-158) | 75 (36-205) | Z=-1.578  p=0.11 |
| First order Energy  Median (Min-Max) | | 35274419(480527-885888062) | 6525208(705863-42805784) | 67389108(480527-885888062) | Z= 5.162  p=0.000 ⃰ |
| First order Entropy  Median (Min-Max) | | 0.87 (0.18-2.62) | 0.86 (0.23-1.05) | 0.89 (0.18-2.62) | Z= -0.708  p= 0.479 |
| First order Interquartile Range  Median (Min-Max) | | 26 (10-71) | 24 (10-33) | 27 (16-71) | Z= 2.527  p= 0.011 ⃰ |
| First order Kurtosis  Median (Min-Max) | | 3.12 (2.23-20.41) | 3.085(2.47-20.41) | 3.128 (2.23-13.42) | Z= .906  p= .365 |
| First order Maximum  Median (Min-Max) | | 140(79-1539) | 124(84-183) | 142(79-1539) | Z= 2.363  p= .018 ⃰ |
| first order Mean Absolute Deviation  Median (Min-Max) | | 16.13 (6.23-94.63) | 14.92 (6.23- 19.05) | 16.16 (10.57- 94.63) | Z= -2.286  p= .022 ⃰ |
| First order Mean  Median (Min-Max) | | 52.09 (14.47-134.79) | 60.554(22.14- 134.71) | 47.87 (14.47-134.79) | Z= -2.574  p=.010 ⃰ |
| First order Median  Mean ±SD | | 54.33±26.41 | 66.71±27.54 | 50.20±24.87 | t= 2.68  p= .009 ⃰ |
| First order Minimum  Median (Min-Max) | | -30(-673- 73) | -8.09(-95- 730) | -34(-678-72) | Z= 2.85  p=.004 ⃰ |
| First order Range  Median (Min-Max) | | 159.5(54-2217) | 149(54-205) | 174.00(101-2217) | Z= 3.43  p=.001 ⃰ |
| First order Robust Mean Absolute Deviation  Median (Min-Max) | | 11.16 (4.35-33.83) | 10.36 (4.35-13.89) | 11.42 (6.62-33.83) | Z= 2.50  p=.012 ⃰ |
| First order Root Mean Squared  Median (Min-Max) | | 55.89 (21.98-179.50) | 65.013 (29.70-136.22) | 53.39 (21.98-179.50) | Z= 2.23  p=.026 ⃰ |
| First order Skewness  Median (Min-Max) | | 0.0093 (-3.31-2.27) | -0.098 (-3.31-0.53) | 0.092 (-0.61-2.27) | Z= 2.899  p=.004 ⃰ |
| First order Total Energy | | 282195352(3844216-7087104496) | 52201664(5646904- 342446272) | 539112864(3844216- 7087104496) | Z= 5.16  p=.000 ⃰ |
| First order Uniformity  Median (Min-Max) | | 0.61(0.27-0.95) | 0.59(0.49- 0.93) | 0.61(0.27-0 .95) | Z= .32  p=.74 |
| First order Variance  Median (Min-Max) | | 418.57(65.67-29009.17) | 392.04(65.67- 628.39) | 421.75(175.26- 29009.17) | Z= 1.97  p=.049 ⃰ |
| GLCM Autocorrelation  Median (Min-Max) | | 4.71(1.72-158.66) | 4.60(1.72- 13.85) | 4.98(2.43- 158.66) | Z= 1.29  p=.19 |
| GLCM Cluster Prominence  Median (Min-Max) | | 0.68 (0.091-6698.28) | 0.57 (0.11-2.39) | 0.69(0.091-6698.28) | Z= 1.85  p=.06 |
| GLCM Cluster Shade  Median (Min-Max) | | 0.087 (-0.70-238.42) | 0.05 (-0.70-0.37) | 0.09 (-0.46- 238.42) | Z= .79  p=.42 |
| GLCM Cluster Tendency  Median (Min-Max) | | 0.48 (0.059-23.94) | 0.45 (0.072-0.755) | 0.48 (0.059-23.94) | Z= .73  p=.47 |
| GLCM Contrast  Median (Min-Max) | | 0.26 (0.034-4.68) | .28 (0.053-0.51) | 0.26 (0.03-4.68) | Z= .22  p=.83 |
| GLCM Correlation  Median (Min-Max) | | 0.18(-0.025-0 .69) | .14 (-0.025-0.52) | .21 (-0.005-0.69) | Z=1.51  p=.13 |
| GLCM Difference Average  Mean ±SD | | 0.22±0.15 | 0.26±0.12 | 0.28±0.16 | t= .46  p= .65 |
| GLCM Difference Entropy  Mean ±SD | | 0.809±0.25 | 0.78±.21 | 0.81±.27 | t= -.42  p= .67 |
| GLCM Difference Variance  Median (Min-Max) | | 0.19 (0.033-3.54) | .20 (0.051- 0.28) | 0.19 (0.033-3.54) | Z=0 .26  p=0.79 |
| GLCM Id  Mean ±SD | | 0.86±0.065 | 0.86± 0.060 | 0.86± 0.067 | t= 0.28  p= 0.78 |
| GLCM Id m  Mean ±SD | | 0.86±0.066 | 0.86± 0.06 | 0.86± 0.068 | t= 0.33  p= 0.74 |
| GLCM I dmn  Median (Min-Max) | | 0.98 (0.907-0 .99) | 0.97 (0.907- 0.99) | 0.98 (0.93- 0.99) | Z=-3.14  p=.002 ⃰ |
| GLCM I dn  Mean ±SD | | 0.94± 0.029 | 0.93± 0.034 | 0.94±0.027 | t= 1.95  p= 0.054⃰ |
| GLCM Imc1  Median (Min, Max) | | -0.048 (-0.33, 0.0025) | -0.047 (-0.20, - 0.005) | -.051(-0.33, -0.0025) | Z= 0.85  p=0.39 |
| GLCM Imc2  Median (Min-Max) | | 0.24 (0.03-0.84) | 0.20(0.069- 0.57) | 0.25(0.036- 0.84) | Z= .94  p=0.35 |
| GLCM Inverse Variance  Median (Min-Max) | | 0.26 (.03- 0.51) | 0.28(0.050- 0.46) | 0.25(0.034- 0.51) | Z= .07  p=0.95 |
| GLCM Joint Average  Median (Min-Max) | | 2.16 (1.28- 12.40) | 2.14(1.28- 3.71) | 2.22(1.52- 12.40) | Z= 1.28  p=0.20 |
| GLCM Joint Energy  Median (Min-Max) | | 0.40 (0.13- 0.92) | 0.40(0.25-0.89) | 0.40(0.13- 0.92) | Z= 0.51  p=0.60 |
| GLCM Joint Entropy  Median (Min-Max) | | 1.66 (0.33- 4.57) | 1.68(0.42- 2.03) | 1.64(.33- 4.57) | Z=0.81  p=0.42 |
| GLCM MCC  Median (Min-Max) | | 0.22 (0.032-0.81) | .21(0.076- 0.52) | 0.22(0.032- 0.81) | Z=0.89  p=0.37 |
| GLCM Maximum Probability  Median (Min-Max) | | 0.59 (0.25-0.95) | 0.59(.29-0.94) | 0.59(0.25- 0.95) | Z= 0.24  p=0.81 |
| GLCM Sum Average  Median (Min-Max) | | 4.32 (2.567- 24.80) | 4.28(2.56- 7.43) | 4.45(3.05- 24.80) | Z= 1.28  p=0.20 |
| GLCM Sum Entropy  Median (Min-Max) | | 1.36 (0.30- 3.46) | 1.35(0.36- 1.63) | 1.39(.30- 3.46) | Z= 0.89  p=0.37 |
| GLCM first order Sum Squares  Median (Min-Max) | | 0.20 (0.023- 7.15) | 0.19(0.03-0 .25) | 0.20(0.023- 7.15) | Z= 0.52  p=0.60 |

**Supplementary table S3: Cross validation metrics (classification performance)**

| Fold | Accuracy  (%) | Precision | Recall | F1-SCORE |
| --- | --- | --- | --- | --- |
| 1 | 77.8 | 0.917 | 0.786 | .85 |
| 2 | 73.8 | 0.846 | 0.786 | .81 |
| 3 | 84.2 | 0.867 | 0.929 | .90 |
| 4 | 77.8 | 0.867 | 0.867 | .87 |
| 5 | 83.3 | 0.929 | 0.867 | .90 |
| Mean | 79.38 | .885 | .847 | .864 |
| SD | 4.32 | .036 | .061 | .034 |
